# Supplementary material for: Vitamin D Modified DSS-Induced Colitis in Mice via STING Signaling Pathway
Source: Biology (Basel). 2025 Jun 18;14(6):715. doi: 10.3390/biology14060715 (PMC12190092; doi:10.3390/biology14060715)
Supplement: Supplementary file 1 [file biology-14-00715-s001.zip › Supplementary Materials S1: 16s-report of gut microbiota/Groups/group_compare.html]

1.0beta6  
Check for updates   
Created & maintained by Francois Bertrand   
Graphic design by Jean-Francois Hains

DataFrame

NO COMPARISON TARGET

20

ROWS

0

DUPLICATES

2.5 kb

RAM

2

FEATURES

1

CATEGORICAL

0

NUMERICAL

1

TEXT

Associations

1

ID

VALUES:

20

(100%)

MISSING:

---

DISTINCT:

20

(100%)

1

5%

-

-

C1

1

5%

-

-

C4

1

5%

-

-

B5

1

5%

-

-

B2

1

5%

-

-

D3

1

5%

-

-

B4

1

5%

-

-

A1

13

65%

-

-

(Other)

2

Groups

VALUES:

20

(100%)

MISSING:

---

DISTINCT:

4

(20%)

Associations

Showing ONLY dataset "DataFrame"
  
• SQUARES are categorical associations (uncertainty coefficient & correlation ratio) from 0 to 1. The uncertainty coefficient is **assymmetrical**,
(approximating how much the elements on the left PROVIDE INFORMATION on elements in the row).
  
• CIRCLES are numerical correlations (Pearson's) from -1 to 1.
  
• The trivial DIAGONAL is intentionally left blank for clarity.

Associations

Showing ONLY dataset "None"
  
• SQUARES are categorical associations (uncertainty coefficient & correlation ratio) from 0 to 1. The uncertainty coefficient is **assymmetrical**,
(approximating how much the elements on the left PROVIDE INFORMATION on elements in the row).
  
• CIRCLES are numerical correlations (Pearson's) from -1 to 1.
  
• The trivial DIAGONAL is intentionally left blank for clarity.


ID

MISSING:

---

1

5%

-

-

C1

1

5%

-

-

C4

1

5%

-

-

B5

1

5%

-

-

B2

1

5%

-

-

D3

1

5%

-

-

B4

1

5%

-

-

A1

1

5%

-

-

C2

1

5%

-

-

A3

1

5%

-

-

D2

1

5%

-

-

C3

1

5%

-

-

C5

1

5%

-

-

A5

1

5%

-

-

B3

1

5%

-

-

A4

1

5%

-

-

D5

1

5%

-

-

B1

1

5%

-

-

A2

1

5%

-

-

D1

1

5%

-

-

D4

Groups

MISSING:

---

TOP CATEGORIES

---

D

5

25%

C

5

25%

B

5

25%

A

5

25%

ALL

20

100%


CATEGORICAL ASSOCIATIONS  
(UNCERTAINTY COEFFICIENT, 0 to 1)

Groups  
PROVIDES INFORMATION ON...

---

  

THESE FEATURES GIVE INFORMATION  
ON Groups:

---

  

NUMERICAL ASSOCIATIONS  
(CORRELATION RATIO, 0 to 1)

Groups  
CORRELATION RATIO WITH...

---
